# Injury incidence and risk factors in youth soccer players: a systematic literature review. Part II: Intrinsic and extrinsic risk factors

**DOI:** 10.5114/biolsport.2023.109962

**Published:** 2022-01-03

**Authors:** Mauro Mandorino, Antonio J. Figueiredo, Masar Gjaka, Antonio Tessitore

**Affiliations:** 1 Department of Movement, Human and Health Sciences, University of Rome “Foro Italico”, Rome, Italy; 2 University of Coimbra, Faculty of Sport Sciences and Physical Education, Research Unit for Sport and Physical Activity, Coimbra, Portugal; 3 Department of Sport and Movement Science, University for Business and Technology, Pristina, Republic of Kosovo

**Keywords:** Youth soccer, Injury, Risk factors, Prevention, Fatigue, Recovery

## Abstract

Injury is defined as a complex phenomenon determined by the non-linear interaction of several intrinsic and extrinsic factors. The objective of the present study was to perform a systematic literature review on the injury risk factors in young soccer players. After electronic database searching, articles in line with the inclusion criteria were selected for the systematic review. Injury risk factor data were extracted and grouped in intrinsic and extrinsic risk factors. The main findings of the current review are as follows: (1) alteration in neuromuscular control such as limb asymmetry and dynamic knee valgus are important intrinsic risk factors; (2) maturation status may impair neuromuscular control and increase the risk of injury; (3) fatigue and poor recovery contribute to the onset of overuse injuries; (4) the impact of anthropometric factors is still unclear; (5) previous injuries increase the risk of re-injuries; (6) highly skilled players are more exposed to risk of injuries; (7) high external workloads increase the risk of injuries; (8) playing position, as well as sport specialization, exposes young soccer players to greater injury risk. Many factors (e.g., neuromuscular control, training load, maturity status) can modify the susceptibility to injury in young soccer players. Being aware of the complex interaction between these factors is essential to identify players at higher risk of injury, develop adequate prevention strategies, and from a scientific point of view move from a reductionist to a complex system approach.

## INTRODUCTION

Sports practice induces several positive effects on both psychological (e.g. self-esteem, emotional regulation, social skills) [1] and physical spheres (e.g. body composition, aerobic fitness, muscular strength) [2]. Nevertheless, despite the numerous health benefits, sports practice may be dangerous in terms of accidents and injuries [3]. Soccer, considered a safe sport [4–6], was reported to have a higher injury rate compared to other team sports, such as rugby, basketball, and American football [7].

Soccer is characterised by high intensity changes of direction, sprints, and jumps [8]. In addition, nowadays soccer matches lead to greater intensity and physical efforts than in the past [9], and consequently more intense training is required to improve the physical fitness demands [10]. These factors expose young soccer players to a higher risk of injuries, compared to adult players. Indeed, in contrast to adults, young athletes experience maturation processes characterised by rapid changes in body size, shape, composition, and hormonal release [11]. All these factors, together with a concomitant alteration in motor coordination and neuromuscular control [12], could increase the susceptibility to injury in young soccer players.

Thus, considering also the negative effects related to youth soccer practice (e.g., dropout, talent development stagnation), it is important to develop prevention strategies. However, the introduction of prevention measures represents the third step of a more complex process known as “the sequence of prevention” [13]. According to this model, the first step consists in identifying and describing sport injuries (e.g., incidence, severity). The second step is to analyse the risk factors and mechanisms underlying injuries, while, the third step allows prevention strategies to be introduced and subsequently their effect on the athletes to be monitored [13]. Regarding the first step, our companion review (part I) [108] aimed to provide epidemiological information of injuries, reporting injury incidence, severity, types, mechanism, and anatomical location. The purpose of this second review (part II) is to identify the injury risk factors.

In general, an injury is a complex phenomenon determined by the interaction of different factors, as explained by Meeuwisse’s dynamic model [14]. According to this model, the risk factors are traditionally classified as intrinsic (e.g. body composition, gender) and extrinsic (e.g. turf type, sports equipment) ones.

The interaction between intrinsic and extrinsic risk factors predis-poses soccer players to injury; however, the presence of an inciting event such as playing position or match schedule represents the real final factor that causes the onset of the injury [14]. Thus, it is unlikely that a single factor is the cause of injury, but rather the interaction between player and environment. Therefore, it is crucial for coaches and physical trainers to be aware of all the injury risk factors, particularly those that could be modified through training and behavioural norms (modifiable risk factors).

Previous authors have already attempted to review injury risk factors in soccer, but these studies are limited to specific injury types such as anterior cruciate ligament tear [15] or hamstring injuries [16].

Therefore, the aim of this review is to identify all the potential injury risk factors and their interactions in young soccer players to provide a general overview for sport practitioners.

## MATERIALS AND METHODS

### Search strategy

We conducted a systematic review of the literature according to the Preferred Reporting Items for Systematic Reviews and Meta-Analyses (PRISMA) statement [17]. The eligible studies were searched by two independent researchers consulting the following electronic databases: ERIC (Educational Resources Information Center), PubMed/ NCBI (National Center for Biotechnology Information, U.S. National Library of Medicine), Scopus, SPORTDiscus via EBSCOhost and Web of Science (WOS), from inception up to the end of February 2021. In each database, the search was performed as follows: [soccer OR football] AND [youth OR young OR adolescen*] AND [injur* OR risk of injury OR impairments].

All the articles were collected using Excel Software (Microsoft Excel 2016, Microsoft Corporation, Washington, USA) to manage duplicates and screening procedures.

### Inclusion and exclusion criteria

The systematic literature review focused on two main topics: injury epidemiological data and injury risk factors in youth soccer players; thus, the inclusion criteria were general and specific for each topic.

General inclusion criteria: (1) published original data (i.e., abstracts, books, reviews, systematic reviews, and meta-analysis were excluded); (2) published in the English language; (3) published in a peer-reviewed scientific journal; (4) articles found in an electronic database up to the 28th of February 2021. Finally, to allow the identification of relevant papers not found during the electronic search, the snowballing technique was applied.

Inclusion criteria for injury epidemiological data: (1) samples of young male and female soccer players (7–18 years old); (2) articles which collected at least one outcome related to injury epidemio-logical data: injury incidence, injury type, severity, re-injury, anatomic location (3) prospective or retrospective studies.

Inclusion criteria for injury risk factors: (1) samples of young male and female soccer players (5–18 years old); (2) articles that analysed risk factors connected to the onset of injury; (3) articles identifying injury predisposition factors (4) prospective, retrospective, cross-sectional studies, randomized control trials (RCT).

**FIG. 1:**
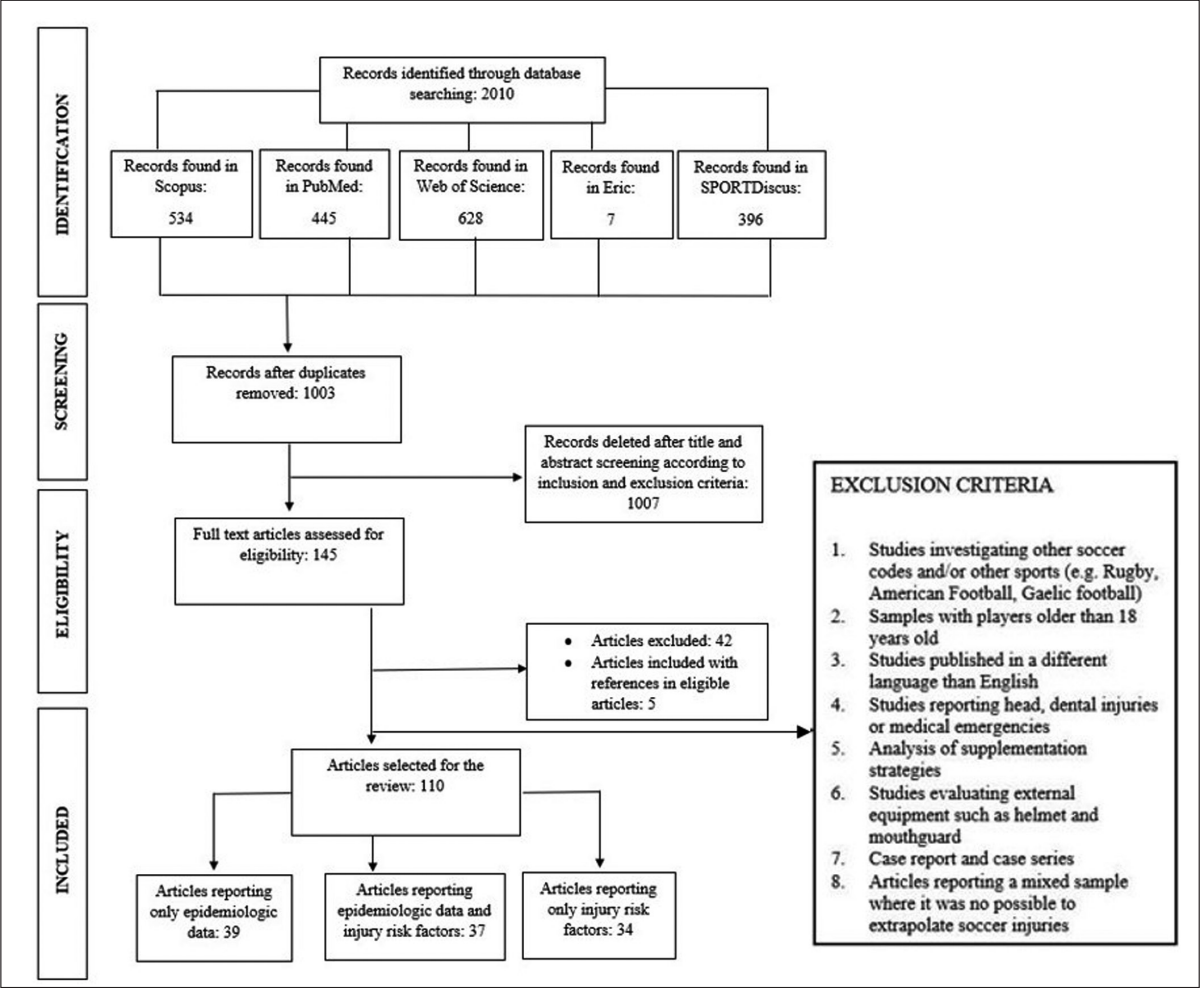
PRISMA Flow Chart.

Exclusion criteria are presented in Figure 1.

### Study selection process

Electronic database searching was initially performed by one reviewer (MM). Then, deletion of duplicates was done by two reviewers (MM and AT). After this step, considering the high amounts of articles identified, a preliminary title screening was conducted, and the selected articles were subjected to abstract screening according to the inclusion criteria previously mentioned.

The full text of the articles identified for eligibility was analysed by three reviewers (AT, MG, MM) for the two main topics: injury epidemiological data and injury risk factors. Thus, the included articles were organised separately according to the area of interest and subjected to the data extraction process conducted by two reviewers (AT, MM).

### Methodological quality of individual studies

Following the same procedure reported by Hume et al. [18], two authors (MM, AT) independently assessed each article reported in the current review using a 6-item custom methodological quality assessment scale. The six items were (P1) study design (0 = cross sectional, retrospective cohort study, 1 = prospective cohort study, randomized-case control, quasi-experimental); (P2) participants’ level (0 = non-elite, 1 = elite/sub-elite); (P3) sample size (0 = less than 20 subjects recruited, 1 = more than 20 subjects recruited); (P4) association with injury onset (0 = not investigated, 1 = investigated); (P5) Number of risk factors investigated (0 = only one, 1 = more than one); (P6) Injury risk results (0 = not clearly reported, 1 = clearly reported and tabulated). The evaluation process together with the final quality score is presented in Table 1. The quality score calculated was not considered as an exclusion criterion.

**TABLE 1:** General information of studies selected

REFERENCES	STUDY DESIGN (QUALITY SCORE)	P1	P2	P3	P4	P5	P6	COUNTRY	DURATION OF DATA COLLECTION	LEVEL OF YOUNG PLAYERS	SEX OF PLAYERS
Aoki et al. [39]	Prospective cohort study (5)	1	0	1	1	1	1	Japan	1 season	Non-elite	Male
Bacon and Mauger [54]	Prospective cohort study (6)	1	1	1	1	1	1	UK	2 seasons	Elite	Male
Bastos et al. [45]	Retrospective study (4)	0	0	1	1	1	1	Brazil	Not available	Non-elite	Male
Bell et al. [67]	Retrospective survey (4)	0	0	1	1	1	1	USA	Not available	Non-elite	Male/Female
Bianco et al. [19]	Prospective cohort study (4)	1	1	1	1	0	0	Italy	1 season	Elite	Male
Bowen et al. [21]	Prospective cohort study (6)	1	1	1	1	1	1	UK	2 seasons	Elite	Male
Brink et al. [5]	Prospective cohort study (6)	1	1	1	1	1	1	The Netherlands	2 seasons	Elite	Male
Bult et al. [43]	Prospective cohort study (5)	1	1	1	1	0	1	The Netherland	3 seasons	Elite	Male
Clausen et al. [63]	Prospective cohort study (5)	1	0	1	1	1	1	Denmark	1 season	Non-elite	Female
Cloke et al. [75]	Prospective cohort study (5)	1	1	1	1	1	0	UK	5 seasons	Elite	Male
Cloke et al. [78]	Prospective cohort study (5)	1	1	1	1	1	0	UK	6 seasons	Elite	Male
De Ridder et al. [36]	Prospective cohort study (6)	1	1	1	1	1	1	Belgium	3 seasons	Elite	Male
De Ste Croix et al. [22]	Quasi-experimental design (4)	1	1	1	0	0	1	UK	Not available	Elite	Female
Engin & Arslan [84]	Retrospective study (5)	0	1	1	1	1	1	Turkey	Not available	Elite	Male
Emery & Meeuwisse [60]	Prospective cohort study (5)	1	0	1	1	1	1	Canada	20 weeks	Non-elite	Male/Female
Emery et al. [33]	Prospective cohort study (5)	1	0	1	1	1	1	Canada	13 weeks	Non-elite	Male/Female
Ferreira et al. [37]	Cross-sectional study (3)	0	1	1	0	1	0	Brazil	Not available	Elite	Male
Frisch et al. [27]	Prospective cohort study (5)	1	0	1	1	1	1	Luxemburg	1 season	Non-elite	Male
Froholdt et al. [50]	Prospective cohort study (5)	1	0	1	1	1	1	Norway	1 season	Non-elite	Male/Female
Frome et al. [68]	Retrospective survey (4)	0	0	1	1	1	1	USA	Not available	Non-elite	Male
Haag et al. [56]	Cross-sectional study (4)	0	0	1	1	1	1	Germany	Not available	Non-elite	Male/Female
Hägglund & Waldén [58]	Prospective cohort study (5)	1	0	1	1	1	1	Sweden	1 season	Non-elite	Female
Hoff & Martin [53]	Retrospective survey (3)	0	0	1	1	0	1	USA	Not available	Non-elite	Male/Female
Isik et al. [73]	Retrospective survey (5)	1	1	1	0	1	1	Turkey	Not available	Elie	Male
John et al. [40]	Cross-sectional study (2)	0	0	1	0	0	1	Germany	Not available	Non-elite	Male
Johnson et al. [46]	Prospective cohort study (5)	1	1	1	1	0	1	UK	6 seasons	Elite	Male
Johnson et al. [35]	Prospective cohort study (6)	1	1	1	1	1	1	UK	2 seasons	Elite	Male
Kemper et al. [25]	Prospective cohort study (6)	1	1	1	1	1	1	The Netherland	1 season	Elite/Non-elite	Male
Ko et al. [34]	Prospective cohort study (5)	1	0	1	1	1	1	USA	1 season	Non-elite	Male
Koenig & Puckree [42]	Cross-sectional study (4)	0	0	1	1	1	1	South Africa	Not available	Non-elite	Female
Kofotolis [24]	Prospective cohort study (5)	1	0	1	1	1	1	Greece	1 season	Non-elite	Male
Kucera et al. [41]	Prospective cohort study (4)	1	0	1	1	0	1	USA	4 seasons	Non-elite	Male/Female
Le Gall et al. [28]	Prospective cohort study (5)	1	1	1	1	0	1	France	10 seasons	Elite	Male
Lehnert et al. [52]	Quasi-experimental design (4)	1	1	0	0	1	1	Czech Republic	Not available	Elite	Male
Lehnert et al. [30]	Quasi-experimental design (5)	1	1	1	0	1	1	Czech Republic	Not available	Elite	Male
Lukasek et al. [44]	Cross-sectional study (1)	0	0	0	0	0	1	Czech Republic	Not available	Non-elite	Male
Materne et al. [86]	Prospective cohort study (5)	1	1	1	1	0	1	Qatar	4 seasons	Elite	Male
Nagle et al. [76]	Prospective cohort study (3)	1	0	1	1	0	0	USA	8 seasons	Non-elite	Male/Female
Nakazawa et al. [74]	Cross-sectional study (2)	0	0	0	1	0	1	Japan	Not available	Non-elite	Male
Namazi et al. [70]	Prospective cohort study (6)	1	1	1	1	1	1	Iran	1 season	Elite	Male
Nguyen et al. [49]	Longitudinal descriptive study (3)	1	0	0	0	1	1	USA	3 seasons	Non-elite	Female
O’Kane et al. [65]	Prospective cohort study (6)	1	1	1	1	1	1	USA	4 seasons	Elite	Female
O’Kane et al. [48]	Prospective cohort study (5)	1	1	1	1	1	1	USA	2 seasons	Elite	Female
O’Kane et al. [61]	Prospective cohort study (6)	1	1	1	1	0	1	USA	2 seasons	Elite	Female
Price et al. [79]	Prospective cohort study (5)	1	1	1	1	1	0	UK	2 seasons	Elite	Male
Räisänen et al. [47]	Prospective cohort study (4)	1	0	1	1	0	1	Finland	1 year	Non-elite	Male/Female
Raya-González et al. [31]	Prospective cohort study (6)	1	1	1	1	1	1	Spain	1 season	Elite	Male
Read et al. [20]	Prospective cohort study (6)	1	1	1	1	1	1	UK	1 season	Elite	Male
Read et al. [23]	Cross-sectional study (4)	0	1	1	0	1	1	UK	Not available	Elite	Male
Read et al. [80]	Cross-sectional and prospective cohort study (6)	1	1	1	1	1	1	UK	1 season	Elite	Male
Rolls and George [72]	Prospective cohort study (6)	1	1	1	1	1	1	UK	1 season	Elite	Male
Rommers et al. [83]	Prospective cohort study (6)	1	1	1	1	1	1	Belgium	1 season	Elite	Male
Rommers et al. [87]	Prospective cohort study (5)	1	1	1	1	0	1	Belgium	2 seasons	Elite	Male
Rosenbaum et al. [77]	Prospective cohort study (4)	1	0	1	1	0	1	USA	2-day tournament	Non-elite	Male/Female
Rossler et al. [59]	Prospective cohort study (5)	1	0	1	1	1	1	Czech Republic and Switzerland	2 seasons	Non-elite	Male/Female
Sanz et al. [81]	Prospective cohort study (5)	1	1	1	1	1	0	Spain	1 season	Elite	Male
Schwebel et al. [29]	Prospective cohort study (4)	1	0	1	1	1	0	USA	8 weeks	Non-elite	Male
Sieland et al. [85]	Prospective cohort study (6)	1	1	1	1	1	1	Germany	2 seasons	Elite	Male
Soligard et al. [26]	Prospective cohort study (4)	1	0	1	1	0	1	Norway	1 season	Non-elite	Female
Steffen et al. [62]	Prospective cohort study (5)	1	0	1	1	1	1	Norway	1 season	Non-elite	Female
Steffen et al. [64]	Prospective cohort study (5)	1	0	1	1	1	1	Norway	8 months	Non-elite	Female
Sugimoto et al. [57]	Cross-sectional study (4)	0	0	1	1	1	1	USA	Not available	Non-elite	Female
Van der Sluis et al. [66]	Prospective cohort study (5)	1	1	1	1	0	1	The Netherlands	3 seasons	Elite	Male
Van der Sluis et al. [38]	Prospective cohort study (5)	1	1	1	1	0	1	The Netherland	3 seasons	Elite	Male
Venturelli et al. [7]	Prospective cohort study (6)	1	1	1	1	1	1	Italy	1 season	Elite	Male
Watson et al., [55]	Prospective cohort study (5)	1	0	1	1	1	1	USA	20 weeks	Non-elite	Female
Watson et al. [69]	Prospective cohort study (5)	1	0	1	1	1	1	USA	20 weeks	Non-elite	Female
Westbrook et al. [82]	Cross-sectional study (3)	0	0	1	0	1	1	USA	Not available	Non-elite	Female
Wollin et al. [51]	Quasi-experimental design (4)	1	1	0	0	1	1	Australia	3 days	Elite	Male
Wright et al. [71]	Quasi-experimental design (4)	1	1	0	0	1	1	UK	Not available	Elie	Female
Zebis et al. [32]	Cluster-randomized controlled trial (4)	1	0	1	1	0	1	Denmark	1 season	Non-elite	Female

## RESULTS

### Search results

Of the 2010 articles found through electronic database searching, only 110 articles met the inclusion criteria (Figure 1). Then, among these, the following articles [5, 7, 19–87] reported injury risk factors and were organised in the present review as follows:

– Intrinsic risk factors (neuromuscular control, physiological and psychological factors, biological and anthropometric factors, previous injuries, technical and tactical skills).
– Extrinsic risk factors (playing surface, shoes, external workload, playing position, sport specialization).

### Injury risk factors

### Intrinsic risk factors

Intrinsic risk factors are defined as individual biological and psychosocial characteristics which make athletes prone to injury [88]. A total of fifty-nine articles [5, 7, 20, 22–27, 29–31, 33–43, 45–52, 55–66, 68–74, 78–84, 86, 87] reported intrinsic injury risk factors.

### Neuromuscular control

Twenty-six articles investigated neuromuscular control in young soccer players [20, 22, 23, 27, 30, 33, 34, 36, 37, 40, 42, 47–49, 51, 52, 58, 61, 70, 71, 73, 74, 78, 79, 82, 84]. Among these, four studies [22, 30, 52, 71] used the SAFT protocol (Prozone) to replicate soccer-specific fatigue and to analyse change in pre- and post-test neuromuscular control. Lehnert et al. [30, 52] in two different studies observed a reduction in both absolute and relative leg stiffness. No changes were observed in isokinetic strength of the hamstrings and quadriceps, or in the hamstring/quadriceps ratio. In one study [52], the authors also reported a decrease in the reactive strength index. De Ste Croix et al. [22] observed an electromechanical delay (EMD) longer in the U13 age group compared with U15 and U17 after the SAFT protocol. However, the same test did not show alteration in knee kinematics during a single leg drop jump and countermovement jump in female soccer players [71].

Instead, Wollin et al. [51] investigated how fatigue, induced by congested soccer matches, impaired neuromuscular control. The authors detected a transient reduction in hamstring strength, with a recovery time needed superior to 48 hours.

De Ridder et al. [36] identified that a low strength level of the posterior chain hip muscle was associated with ankle sprains, and similarly low hip and knee muscle strength was significantly related to lower limb injuries [70]. Regarding this issue, Nguyen et al. [49] were interested in investigating whether changes in the hip strength occurred when youth female soccer players increased in age. The authors did not find changes in isometric hip strength but in the hip range of motion, particularly, they found an increase in passive hip abduction and in passive hip internal rotation.

Four studies [47, 48, 61, 82] investigated knee kinematics during a landing task [48, 61] and during single leg squat [47]. O’Kane et al. [61] found that the normalised knee separation (NKS) on landing that was in the ≤ 10^th^ percentile increased the risk of knee injury in female soccer players. The results are in agreement with a previous study [48], which however found an association between NKS and knee injury, but only in postmenarchal players. Similarly, Westbrook et al. [82] found a greater peak knee abduction angle during a double-leg countermovement jump task in post-pubertal female players. However, Räisänen et al. [47] did not identify the frontal knee projection angle (FKPA) during a single leg squat as an injury risk factor.

Read et al. [20] revealed single leg countermovement jump (SLCMJ) landing force asymmetry as the most relevant injury risk factors, without significant difference for the single leg hop for distance (SLHD) and tuck jump assessment (TJ). However, SLHD was identified as a potential risk factor by Sieland et al. [85]. Static and dynamic balance tests [27, 33, 42] were not sensitive to identify injury risk conditions. Furthermore, three studies [47, 78, 79] found most of the injuries located in the dominant leg. In contrast, Hägglund & Waldén [58] reported a higher prevalence of ACL injuries in the non-dominant leg.

Five studies [34, 40, 74, 80, 84] used a functional test to evaluate neuromuscular control. First, Ko et al. [34] identified the posterior-lateral and posterior-medial score of the star excursion balance test (SEBT) as associated with risk of injury. Similarly, John et al. [40] and Read et al. [80] performed the Y-Balance test (YBT) with young male soccer athletes. Read et al. [80] found a significant association between injury and anterior reach scores. Then, John et al. [40] found a lower score in players with higher maturity offset; however, they did not investigate the association between the test and risk of injuries. Nakazawa et al. [74] found a difference between injured and non-injured groups in the sideways bench test. Finally, Engin & Arslan [84] investigated the association between functional movement screening (FMS) score and risk of musculoskeletal injuries without finding any significant association.

### Physiological and psychological risk factors

Thirteen articles [5, 7, 22, 27, 30, 31, 33, 51, 52, 55, 63, 64, 69] investigated physiological and psychological risk factors.

Five studies [22, 30, 51, 52, 71] focused on the effect of fatigue on physiological parameters. In general, fatigue produced a worsening in hamstring activation [22], reactive strength and stiffness [30, 52], and isometric hamstring strength [51]. Moreover, physical fatigue, assessed through a questionnaire [27], was associated with injuries (HR = 2.32) in young players.

Three studies [5, 31, 69] evaluated the stress state resulting from training and the link with injuries. Brink et al. [5] found an association between internal load (S-RPE), monotony, and strain with traumatic injuries. Furthermore, several subscales of the REST-Q questionnaire, reported in Table 2, were related to illness risk. Likewise, Watson et al. [69] found an association between training load and risk of injuries and illnesses. In contrast, Raya-González et al. [31] did not find a significant association between weekly training load and risk of injuries.

**TABLE 2:** Summary of the risk factors related to injuries in young soccer players.

STUDY	RISK FACTORS EXAMINED	SIGNIFICANT RISK FACTOR IDENTIFIED	STATISTICAL RESULTS	NON-SIGNIFICANT RISK FACTORS	SAMPLE SIZE AND INJURY RATE	MAIN TOPIC AREA
Read et al. [20]	- Chronological age - Maturity offset - Neuromuscular control test: • single leg hop for distance (SLHD) • 75% of maximum hop and stick (75%Hop) • single leg countermovement jump (SLCMJ) • tuck jump assessment (TJ)	1. SLCMJ peak landing vertical ground reaction force asymmetry 2. Lower right leg SLCMJ peak landing vertical ground reaction force relative to body weight 3. Maturational Offset 4. Advanced chronological age	1. U11-U12’s OR = 0.90 *p* = 0.04 U15-U16s OR = 0.91 *p* < 0.001 2. U15-U16’s OR = 0.36 *p* = 0.03 3. U13-U14’s OR = 0.58 *p* = 0.04 4. U18’s OR = 3.62 *p* = 0.04	- single leg hop for distance (SLHD) - 75% of maximum hop and stick (75%Hop) - tuck jump assessment (TJ)	356 elite male youth soccer players were monitored for 10 months (99 sustained a non-contact lower extremity injury)	**NEUROMUSCULAR CONTROL**

Sieland et al. [85]	- Age - Drop Jump - Countermovement jump - Single-leg hop for distance - Side asymmetry-single-leg hop for distance - Sit-and-Reach - Knee extension - Knee flexion - Sprint 10 m - Sprint 30 m - Zig-Zag test without ball - Zig-Zag test with ball	1. Age 2. Side asymmetry-single-leg hop for distance	1. Age *r* = 0.191 *p* = 0.006 2. Side asymmetry-single-leg hop for distance *p* = 0.08	- Drop Jump - Countermovement jump - Single-leg hop for distance - Sit-and-Reach - Knee extension - Knee flexion - Sprint 10 m - Sprint 30 m - Zig-Zag test without ball - Zig-Zag test with ball	93 players were followed during two seasons (125 injuries were recorded)

Engin & Arslan [84]	- FMS score - Asymmetry - Age	1. Age	1. Age OR = 1.57 *P* = 0.002	- FMS score - Asymmetry	57 elite male youth soccer players (27 musculoskeletal injuries were recorded)	**NEUROMUSCULAR CONTROL**

De Ste Croix et al. [22]	- Neuromuscular control of knee after soccer-specific fatigue in elite female youth soccer: - Surface electromyography (EMD) was used to investigate effect of fatigue on hamstrings during eccentric actions at 60, 120 and 180°/s	1. EMD was significantly longer in U13 age group compared with U15 and U17	1. 158 ± 66 ms vs 113 ± 39 *p* = 0.021 (U13vsU15) 158 ± 66 ms vs 120 ± 40 *p* = 0.021 (U13 vs U17)	- No significant main effects for the different muscles (semitendinosus, biceps femoris and gastrocnemius) - No significant main effect for the different velocity (60, 120 and 180°/s)	36 young female soccer players participated to the study	

Read et al. [23]	- Biological maturation (intrinsic risk factors) - Single leg countermovement jump (SLCMJ) height (intrinsic risk factors) - Peak vertical landing forces (pVGRF) (intrinsic risk factors) - Knee valgus (intrinsic risk factors) - Trunk side flexion (intrinsic risk factors)	1. Vertical jump height and absolute p VGRF increased linearly with each stage of maturation 2. Higher landing forces were recorded on the left leg in circa versus post-PHV 3. Significantly less knee valgus was found in post-PHV compared to pre-PHV on the left leg 4. Post-PHV players displayed greater lateral trunk flexion angles on the left leg compared to circa-PHV	1. Cohen’s d effect sizes = 0.85–2.35 *p* < 0.001 2. *d* = -0.40 *p* < 0.05 3. *d* = 0.67 *p* < 0.05 4. *d* = 0.85 *p* < 0.05	- No differences were shown for knee valgus on the right leg	400 elite male youth soccer players took part in the study

Read et al. [80]	- Age - Height - Y-balance score	1. Y-balance Left (%leg length) 2. Y-balance right 3. Age	1. Y-balance Left (%leg length) OR = 0.94, 95% CI 0.91 to 0.98, *p* < 0.001 2. Y-balance Right OR = 1.06, 95% CI 1.0 to 1.10, *P* < 0.05 3. Age OR = 1.49, 95% CI 1.04 to 2.13, P < 0.05	- Height	346 elite male youth soccer players.	**NEUROMUSCULAR CONTROL**

Lehnert et al. [30]	- The parameters were assessed after SAFT protocol: • Reactive strength (RSI) • Absolute leg stiffness • Relative leg stiffness - Isokinetic strength	1. Reactive strength (RSI) 2. Absolute leg stiffness 3. Relative leg stiffness	1. % change = 7.5 *p* < 0.01 2. % change = -8.1 *p* < 0.01 3. % change = -6.4 *p* < 0.01	- Isokinetic strength	20 elite youth soccer players took part in the study

O’Kane et al. [48]	- Normalized knee separation during drop jump (NKS) at prelanding, landing and take-off	Among postmenarchal players: 1. NKS ≤ 10th percentile (most extreme valgus) at prelanding and landing, significantly increased lower extremity injury 2. NKS ≤ 10th percentile (most extreme valgus) at prelanding and landing, significantly increased knee injury 3. NKS ≤ 10th percentile (most extreme valgus) during take-off, significantly increased lower extremity injury 4. NKS ≤ 10th percentile (most extreme valgus) during take-off, significantly increased knee injury	1. RR = 1.92 95% CI 1.17 to 3.15 2. RR = 3.62 95% CI 1.18 to 11.09 3. RR = 1.80 95% CI 1.01 to 3.23 4. RR = 1.66 95% CI 1.04 to 2.64	- Among premenarchal players, there was no statistically significant association between NKS at prelanding, landing and take-off and the risk of lower extremity or knee injury	351 female youth soccer players were followed for 1 season (173 lower extremity injuries, with 43 knee injuries, were recorded)	**NEUROMUSCULAR CONTROL**

Ko et al. [34]	- Age - Height - Body mass - BMI% - Star Excursion Balance Test (SEBT): • Anterior reach direction (AN) • Posterior-medial reach direction (PM) • Posterior-lateral (PL) reach direction - Single-Leg Hop Test (SLHT)	1. SEBT-PM 2. SEBT-PL 3. SLHT	1. OR = 16.61 Cohens *d* = 1.15 *p* < 0.05 2. OR = 20.88 Cohens *d* = 1.31 *p* < 0.05 3. OR = 32.53 Cohens *d* = 1.20 *p* < 0.05	- Age - Height - Body mass - BMI% - SEBT-AN	64 players were followed during one season (12 players sustained ankle sprain)	**NEUROMUSCULAR CONTROL**

De Ridder et al. [36]	- Body size dependencies and anterior chain/horizontal plan hip muscle forces - Posterior chain hip muscle forces - Hip adduction muscle force - Hip abduction muscle force	1. Higher posterior chain hip muscle forces produced significant reduction in ankle sprains	1. HR = 0.331 95% CI 0.123 to 0.890 *p* < 0.05	- Body size dependencies and anterior chain/horizontal plan hip muscle forces - Hip adduction muscle force - Hip abduction muscle force	133 male youth soccer players were followed over 3 seasons (12 players sustained a lateral ankle sprain)	

Räisänen et al. [47]	- Frontal plane knee projection angle (FPKPA) during the single-leg squat			- Frontal plane knee control in the single-leg squat was not associated with lower extremity injuries	558 players were recruited and monitored for 20 weeks (285 acute lower extremity injuries were reported)

Lehnert et al. [52]	- Reactive strength index (RSI) - Leg stiffness - Isokinetic dynamometry with integrated sEMG	1. RSI 2. Absolute leg stiffness 3. Relative leg stiffness 4. Muscle activation decreased in Rectus femoris, vastus medialis, semimembranosus	1. ES = 0.40 *p* < 0.05 2. ES = 0.55 *p* < 0.05 3. ES = 0.68 *p* < 0.05 4. ES = 0.33–0.97 *p* < 0.05	- H/Q FUNC did not change - Muscle activation of vastus lateralis and biceps femoris did not change	18 elite youth soccer players took part in the study	**NEUROMUSCULAR CONTROL**

Koenig & Puckree [42]	- Sway index (SI) - Limits of stability direction control (LOS)			- Sway index (SI) Limits of stability direction control (LOS)	80 adolescent female soccer players took part in the study	

Nguyen et al. [49]	- Hip range of motion (ROM): • Passive hip internal rotation (IR) • Passive hip external rotation (ER) • Passive hip abduction (ABD) • Passive hip adduction (ADD) - Isometric hip strength: • Isometric hip abduction (ABD) • Isometric hip extension (EXT)	1. Hip ABD ROM significantly increased 2. Hip ADD ROM significantly decreased 3. Hip IR ROM increased 4. Hip ER ROM decreased	*1. p* < 0.001 *2. p* = 0.009 *3. p* = 0.001 *4. p* < 0.001		- Isometric hip ABD Isometric hip EXT	14 female youth soccer players were recruited in the study

John et al. [40]	- Balance error scoring system (BESS) - Y-Balance test (YBT) • Anterior reach direction • Posteromedial reach direction - Posterolateral reach direction	1. Total BESS score was significantly lowest in participants with the lowest maturity offset 2. Anterior reach direction of the right leg was lower in players with higher maturity offset 3. Anterior reach direction of the left leg was lower in players with higher maturity offset 4. Posteromedial reach direction of the left leg was lower in players with higher maturity offset	1. b = 2.195 *p* = 0.022 2. b = -0.022 *p* = 0.023 3. b = -0.024 *p* = 0.015 4. b = -0.29 *p* = 0.020	- Posterolateral of right and left leg reach direction of YBT - Posteromedial of right direction of YBT	101 male youth soccer players were recruited	**NEUROMUSCULAR CONTROL**

O’Kane et al. [61]	- Joint hypermobility - Hamstring strength - Quadriceps strength - Hip Strength - Normalized knee separation during drop-jump test (NKS) - Playing in another soccer team - Position played in the last month - Participation in another sports team	1. NKS on landing in the ≤ 10th percentile (most valgus appearing) increased lower extremity injuries 2. NKS on landing in the ≤ 10th percentile (most valgus appearing) increased knee injury 3. Playing in another soccer team increased knee overuse injury	1. RR = 2.24 95% CI 1.20 to 4.19 2. RR = 3.2 95% CI 1.52 to 6.71 3. RR = 2.5 95% CI 1.08 to 5.35	- Increase in hamstring strength reduced overuse knee injury - Increase in quadriceps strength reduced overuse knee injury - Increase in hip flexor and external rotation reduced overuse knee injury - Joint hypermobility - Position played in the last month - Participation in another sports team	351 players were monitored over 4 seasons (83 lower extremity overuse injuries were recorded)	**NEUROMUSCULAR CONTROL**

Wollin et al. [51]	- Hamstring maximum voluntary isometric contraction (MVIC) - Perceived pain on isometric hamstring MVIC - Ankle dorsiflexion - Hip extension range of motion - Active knee extension (AKE) - Prone knee bend (PKB)	1. Hamstring strength significantly reduced 24h post-match 1 2. Hamstring strength significantly reduced 48h post-match 1 3. Hamstring strength significantly reduced 24h post-match 2 4. Pain increased moderately 24h post-match 1 5. Pain increased moderately 48h post-match 1 6. PKB reduced 24h post-match 1 7. PKB reduced 24h post-match 2 8. PKB reduced 48h post-match 2	1. Mean difference = – ---0.19 Nm/Kg *p* = 0.001 2. Mean difference = -----0.16 Nm/kg *p* = 0.002 3. Mean difference = -----0.17 Nm/kg *p* = 0.012 4. ES = 0.42 *p* = 0.02 5. ES = 0.38 *p* = 0.036 6. Mean difference = -----2.7° *p* = 0.002 7. Mean difference = -----1.5° *p* = 0.044 8. Mean difference = -----2.7° *p* = 0.023	- Hamstring strength did not change 48 and 72h post-match 2 - There was no change in pain on match day 2, at 24, 48 and 72 post-match 2 None of the other ROM outcome measures demonstrated a significant change during the 2-match congestion period	15 male elite youth soccer players took part in the study	**NEUROMUSCULAR CONTROL**

Brink et al. [5]	- Internal load (Duration × RPE) (S-RPE) - Monotony - Strain - Recovery-stress state (REST-Q) • General stress • Sport-specific stress • General recovery - Sport-specific recovery	1. Duration (Traumatic injury) 2. Duration (Illness) 3. Load (Traumatic injury) 4. Monotony (Traumatic injury) 5. Strain (Traumatic injury) 6. REST-Q: emotional stress (Illness) 7. REST-Q: Social stress (Illness) 8. REST-Q: Conflicts/pressure 9. REST-Q: fatigue (Illness) 10. REST-Q: Lack of energy (Illness) 11. REST-Q: Physical complaints (Illness) 12. REST-Q: Social recovery (Illness) 13. REST-Q: General well-being (Illness) 14. REST-Q: Sleep quality (Illness) 15. REST-Q: Disturbed breaks (Illness) 16. REST-Q: Emotional exhaustion (Illness) 17. REST-Q: Being in shape (Illness) 18. REST-Q: Fitness/injury (Traumatic injury) 19. REST-Q: Fitness/injury (Overuse injury)	1. OR = 1.14 95% CI 1.06 to 1.23 *p* < 0.05 2. OR = 1.12 95% CI 1.00 to 1.26 *p* < 0.05 3. OR = 1.01 95% CI 1.00 to 1.02 *p* < 0.05 4. OR = 2.59 95% CI 1.22 to 5.50 *p* < 0.05 5. OR = 1.01 95% CI 1.00 to 1.01 *p* < 0.05 6. OR = 2.27 95% CI 1.43 to 3.61 *p* < 0.01 7. OR = 2.59 95% CI 1.22 to 5.50 *p* < 0.01 8. OR = 1.69 95% CI 1.18 to 2.42 *p* < 0.01 9. OR = 1.48 95% CI 1.05 to 2.09 *p* < 0.05 10. OR = 1.92 95% CI 1.27 to 2.91 *p* < 0.01 11. OR = 1.88 95% CI 1.24 to 2.83 *p* < 0.01 12. OR = 0.66 95% CI 0.47 to 0.94 *p* < 0.05 13. OR = 0.57 95% CI 0.39 to 0.83 *p* < 0.01 14. OR = 0.58 95% CI 0.40 to 0.83 *p* < 0.01 15. OR = 1.51 95% CI 1.03 to 2.22 *p* < 0.05 16. OR = 1.47 95% CI 1.06 to 2.03 *p* < 0.05 17. OR = 0.56 95% CI 0.40 to 0.79 *p* < 0.01 18. OR = 1.29 95% CI 1.01 to 1.66 *p* < 0.05 19. OR = 1.46 95% CI 1.09 to 1.96 *p* < 0.05	- Duration (Overuse) - Load (Overuse injury, Illness) - Monotony (Overuse injury, Illness) - Strain (Overuse injury, Illness) - REST-Q: the remaining subscales did not show any significant difference	53 elite soccer players were monitored over 2 seasons (320 injuries and 82 illness occurred)	**PHYSIOLOGICAL AND PSYCHOLOGICAL RISK FACTORS**

Watson, et al. [69]	- Subjective well-being • Fatigue • Mood • Stress • Soreness • Sleep quality • Sleep hours - Training load (TL) • Daily • Prior day • Weekly • Monthly - Acute:Chronic	1. Mood (injuries) 2. Daily TL (injuries) 3. Prior day TL (injuries) 4. Weekly TL (illness) 5. Monthly TL (Illness)	1. OR = 0.12 95% CI 0.02 to 0.66 p = 0.011 2. OR = 1.98 95% CI 1.43 to 2.78 *p* < 0.001 3. OR = 1.38 95% CI 1.01 to 1.88 *p* = 0.040 4. OR = 1.50 95% CI 1.13 to 2.00 *p* = 0.005 5. OR = 1.54 95% CI 1.13 to 2.12 *p* = 0.007	- Fatigue - Stress - Soreness - Sleep quality - Sleep hours - Weekly TL (injuries) - Monthly TL (injuries) - Acute:Chronic TL (injuries) - Prior day TL (Illness) - Acute:Chronic TL (Illness)	75 youth female soccer players were monitored through 20-week season (36 injuries and 52 illness were recorded)	**PHYSIOLOGICAL AND PSYCHOLOGICAL RISK FACTORS**

Raya-González et al. [31]	- Sum of intrinsic training load for each week (WL) - Acute chronic workload ratio			- Sum of intrinsic training load for each week (WL) (intrinsic risk factor) Acute chronic workload ratio (intrinsic risk factor)	21 players were followed during one season (27 non-contact injuries were recorded)	

Steffen et al. [64]	- Age - Height - Weight - BMI - Previous injury - Years of organized soccer play - Perception of success - Motivation climate - Life event - Sport anxiety - Brief cope	1. Previous injury 2. Years of organized soccer play 3. High level of perceived life stress 4. High level of perceived mastery climate 5. High level of life event	1. OR = 1.9 95% CI 1.4 to 2.5 *p* < 0.001 2. RR = 1.12 95% CI 1.04 to 1.22 *p* = 0.003 3. OR = 1.7 95% CI 1.3 to 2.2 *p* < 0.001 4. OR = 1.34 95% CI 1.04 to 1.72 *p* = 0.03 5. OR = 1.03 95% CI 1.01 to 1.05 *p* = 0.02	- Age - Height - Weight - BMI - Perception of success - Sport anxiety - Brief cope	1430 youth female players were followed during one season (380 injuries were recorded)

Frisch et al. [27]	- Injury history - Physical fatigue - Emotional stress - Joint laxity - Anthropometric data - Hop for distance test (intrinsic factor) - Aerobic fitness - Maximal isokinetic tests - Static balance test - Dynamic balance test - Squat jump - Countermovement jump	1. Physical fatigue	1. HR = 2.32 95% CI 1.07 to 5.05 *p* = 0.034	- Injury history (intrinsic risk factor) - Emotional stress (intrinsic risk factor) - Joint laxity (intrinsic risk factor) - Anthropometric data (intrinsic risk factor) - Hop for distance test (intrinsic factor) - Aerobic fitness (intrinsic risk factor) - Maximal isokinetic tests (intrinsic risk factor) - Static balance test (intrinsic risk factor) - Dynamic balance test (intrinsic risk factor) - Squat jump (intrinsic risk factor) - Countermovement jump (intrinsic risk factor)	67 players were monitored during one season (163 injuries were registered)	**PHYSIOLOGICAL AND PSYCHOLOGICAL RISK FACTORS**

Kemper et al. [25]	- Body mass - Height - Fat percentage - Growth in height (cm) - Alteration in body mass index (kg/m2) Fat percentage and fat free mass index (kg/m2)	1. Growth rates of at least 6 cm/month 2. Monthly BMI-increase of > 0.3 kg/m2 3. Decrease in BMI-value of at least 0.4 kg/m2	1. OR = 1.63 95% CI 1.06–2.52 *p* = 0.03 2. OR = 1.61 95% CI 1.04–2.49 *p* = 0.03 3. OR = 1.97 95% CI 1.18–2.76	- High fat percentage Fat free mass index (FFMI)	101 male youth players were followed during one season (134 injuries occurred. The injury incidence was 5.9/1000 hours)	**BIOLOGICAL AND ANTHROPOMETRIC FACTORS**

Materne et al. [86]	- Maturity status	1. Early-maturing vs normal-maturing players 2. Early-maturing vs mature players	1. HR = 1.26 95% CI, 1.11–1.42 P < 0.001 2. HR = 1.35 95% CI, 1.17–1.56 P < 0.001		283 male elite soccer player were monitored during four consecutive seasons (a total of 1565 injuries were recorded)	

Johnson et al. [35]	- Maturity timing: • Pre-PHV • Circa-PHV • Post-PHV - Maturity status: • Early-maturer • On-time - Late-maturer	1. Circa-PHV versus Pre-PHV 2. Early/circa-PHV versus on-time/late/pre-PHV 3. On-time/late/circa-PHV versus on-time/late/pre-PHV	1. RR = 2.15 95% CI 1.37 to 3.38 *p* < 0.001 2. RR = 2.42 95% CI 1.22 to 4.81 *p* = 0.01 3. RR = 2.50 95% CI 1.53 to 4.07 *p* = 0.0003	- All other comparisons did not demonstrate significant differences	76 male young soccer players were monitored over two different seasons (88 injuries were recorded)	

Van der Sluis et al. [38]	Maturity timing (PHV)	1. Players who had their PHV at an older age had a higher incidence of overuse injuries before PHV 2. Players who had their PHV at an older age had a higher incidence of overuse injuries during PHV	1. U = 49.50 *r* = 0.40 *p* < 0.05 2. U = 50.5 *r* = 0.35 *p* < 0.05	All other comparisons did not demonstrate significant differences	26 youth male soccer players were followed over 3 years around Peak Height Velocity (39 traumatic and 28 overuse injuries were recorded)

Bult et al. [43]	- Maturity timing (PHV)	1. The injury incidence density (IID) in PHV period 4+5 (6 months after PHV) was significantly higher 2. The IID in PHV period 1 was significantly lower	1. IR = 1.31 95% CI 1.00 to 1.71 2. IR = 0.77 95% CI 0.62 to 0.95	- The IDD for PHV periods 2,3 and 6 were not significantly different when compared with the overall mean	170 players were monitored over 3 seasons (393 acute and 135 overuse injuries were recorded)

Johnson et al. [46]	- Maturity status • Early-maturer • On-time - Late-maturer			- The analysis showed that the injury incidents did not differ significantly between categories of maturity status when adjusted for playing time, mean training time, mean height and position played	292 schoolboy players were followed over six years (476 injuries were registered across all the age groups)

Bastos et al. [45]	- Age - Weight - Height - Body Mass - Duration of training	1. Players taller than 1.67 m reported more injuries than those with 1.66 m 2. Players with more than five years of training reported more injuries than those with less than five years of training	*1. p* = 0.01 *2. p* = 0.003	- Age - Weight - Body Mass	301 athletes were involved in the study	**BIOLOGICAL AND ANTHROPOMETRIC FACTORS**

Rommers et al. [87]	- Growth velocity (cm/y)	1. Growth velocity on injury occurrence demonstrated a 15% increase in injury risk per cm of growth per year	1. OR = 1.15 95%CI: 1.05–1.26 *p* = 0.003		378 male players were involved in the study (105 injuries were recorded)	

Watson et al. [55]	- Age - Body mass - Soccer experience - VO_2max_ ml/kg/min	1. VO_2max_ ml/kg/min	1. OR = 0.94 95% CI 0.90 to 0.98 *p* = 0.009	- Age - Body mass - Soccer experience	54 female adolescent players were followed over 20-week (28 injuries and 38 illnesses were recorded)

Hägglund, & Waldén [58]	- Age - Relative age - BMI - Menarche - Previous acute knee injury - Current knee complaints - Familial disposition ACL injury - Training session per week - Match exposure ratio - Match with another team - Artificial turf exposure	1. Age > 14 years 2. Current knee complaints 3. Familial disposition ACL injury	1. HR = 1.82 95% CI 1.13 to 2.92 *p* = 0.014 2. HR = 2.06 95% CI 1.30 to 3.26 *p* = 0.002 3. HR = 1.87 95% CI 1.15 to 3.03 *p* = 0.012	- Relative age - BMI - Menarche - Previous acute knee injury - Training session per week - Match exposure ratio - Match with another team - Artificial turf exposure	4556 young players were studied (96 acute knee injuries were recorded, 21 of them ACL injuries)	

Rössler et al. [59]	- Age - Sex - Body height percentile category - Body mass percentile category - BMI percentile category - Match-training ratio - Playing position - Foot preferred - Playing surface	1. Body height percentile category (acute injuries) 2. Body height percentile category (overuse injuries) 3. Match training ratio (Match injuries) 4. Left foot preferred (Training injuries) 5. Artificial turf 6. Indoor	1. HR = 1.16 95% CI 1.02 to 1.32 *p* = 0.019 2. HR = 1.21 95% CI 1.03 to 1.42 *p* = 0.026 3. HR = 0.32 95% CI 0.23 to 0.46 *p* < 0.001 4. HR = 1.53 95% CI 1.07 to 2.19 *p* < 0.021 5. RR = 1.39 95% CI 1.12 to 1.73 *p* < 0.001 6. RR = 0.68 95% CI 0.52 to 0.88 *p* < 0.001	- Age - Sex - Body mass percentile category - BMI percentile category - Playing position	A total of 6038 players were followed through one season (417 injuries occurred)	

Sugimoto et al. [57]	- Age - Height - Weight - BMI - Playing position - Hours of training per week - Seasons participated - Muscular strength - Joint laxity Previous injuries	1. Age 2. Weight 3. BMI	1. OR = 1.602 95% CI 1.165 to 2.202 *p* = 0.004 2. OR = 0.908 95% CI 0.834 to 0.989 *p* = 0.026 3. OR = 1.430 95% CI 1.074 to 1.904 *p* = 0.014	- Height - Playing position - Hours of training per week - Seasons participated - Muscular strength - Joint laxity Previous injuries	160 young female soccer players participated in the study	

Froholdt et al. [50]	- Sex - Age	1. Age (older players showed higher injury incidence)	1. RR = 1.7 95% CI 1.3 to 2.2	- Sex	159 were followed throughout 1 season (200 injuries were recorded)

Van der Sluis et al. [66]	- Maturity timing (PHV) • Pre-PHV • PHV • Post-PHV	1. PHV versus Pre-PHV (traumatic injuries)	1. *d = 0.50* *p* = 0.006	- The other comparisons did not show any significant difference	26 young soccer players were monitored over 3 years (178 injuries were recorded)	

Soligard et al. [26]	- Soccer skills: • Technical (ball receiving, passing and shooting, heading, dribbling, tackling) • Tactical (decision-making in ball possession, decision-making not in ball possession, decision-making in defense) • Physiological (endurance, speed/agility, strength, coordination/balance)	Data refer to impact of injury risk factors on overall injuries: 1. Ball receiving 2. Passing and shooting 3. Heading 4. Tackling 5. Decision-making when in ball possession 6. Decision-making when in defence 7. Strength	1. RR = 1.55 95% CI 1.04 to 2.31 2. RR = 1.82 95% CI 1.26 to 2.63 3. RR = 1.50 95% CI 1.13 to 2.00 4. RR = 1.70 95% CI 1.18 to 2.45 5. RR = 1.62 95% CI 1.08 to 2.45 6. RR = 1.81 95% CI 1.23 to 2.65 7. RR = 1.62 95% CI 1.18 to 2.22	- Dribbling - Decision-making when not in ball possession - Endurance - Speed/agility - Coordination/balance	1034 were included in the study (259 injuries were recorded)	**TECHNICAL AND TACTICAL SKILLS**

Venturelli et al. [7]	- Age-category - Field position - Height - Body Mass - BMI - Yo-yo test - Squat jump (SJ) - Countermovement jump (CMJ) - Percentage difference between the two types of jumps (ΔJH) - Sit and reach score - Previous thigh strain injuries	1. Previous injuries 2. ΔJH 3. Height	1. HR = 2.80 CI 95% 1.19 to 6.54 2. HR = 0.79 CI 95% 0.71 to 0.87 3. HR = 1.17 CI 95% 1.06 to 1.25	- Age-category - Field position - Body Mass - BMI - Yo-yo test - Squat jump (SJ) - Countermovement jump (CMJ) - Sit and reach score	96 players were followed during an entire season (27 muscular strain were recorded)	**PREVIOUS INJURIES**

Emery et al. [33]	- Age - Previous injury, past 6 week - Previous injury, past 1 year - Height - Weight - Body mass index - Vertical jump - Predicted VO2max - Eyes closed dynamic balance	1. Female U14 age group 2. Male U14 age group 3. Previous injury, past 1 year	1. RR = 3.13 95% CI 1.14 to 10.67 *p* < 0.05 2. RR = 2.45 95% CI 0.95 to 7.05 *p* < 0.05 3. RR = 1.74 95% CI 1.0 to 3.1 *p* < 0.05	- Height - Weight - Body mass index - Vertical jump - Predicted VO2max Eyes closed dynamic balance	344 were monitored for 13 weeks (78 injuries were reported)	

Clausen et al. [63]	- Previous knee injury - Knee injury and osteoarthritis outcome score (KOOS): • function in daily living (ADL) subscale • Pain subscale • Function in sport and recreation subscale (Sport/Recreation) • Knee-related quality of life subscale (QOL) • Other symptoms subscale	1. Previous knee injury 2. ADL score less than 80 points 3. Sport/recreation score less than 80 points 4. QOL score less than 80 points	1. RR = 3.64 95% CI 1.73 to 7.66 *p* < 0.001 2. RR = 5.00 95% CI 1.53 to 16.38 *p* < 0.001 3. RR = 2.23 95% CI 1.01 to 4.91 *p* < 0.001 4. RR = 3.01 95% CI 1.13 to 8.00 *p* < 0.001	- Pain subscale - Other symptoms subscale	326 young soccer players were included in the study (34 knee injuries were recorded)

Kucera et al. [41]	- One previous injury Two or more previous injuries	1. One previous injury 2. Two or more previous injuries	1. RR = 2.57 95% CI 2.00 to 3.29 2. RR = 2.97 95% CI 2.28 to 3.86		1483 soccer players were monitored over 3 seasons	**PREVIOUS INJURIES**

Steffen et al. [62]	- Age - Height - Weight - Body mass index - Weekly sports participation - Previous injury - Previous ankle injury - Previous knee injury - Previous thigh injury - Previous groin injury Years of organized soccer play	1. Previous injury 2. Previous ankle injury 3. Previous knee injury 4. Previous groin injury 5. Years of organized soccer play	1. OR = 1.9 95% CI 1.4 to 2.5 *p* < 0.001 2. RR = 1.2 95% CI 1.1 to 1.3 *p* < 0.001 3. RR = 1.4 95% CI 1.2 to 1.6 *p* < 0.001 4. RR = 1.6 95% CI 1.2 to 2.1 *p* = 0.004 5. RR = 1.12 95% CI 1.04 to 1.22 *p* = 0.003	- Age - Height - Weight - Body mass index - Weekly sports participation - Previous thigh injury	1430 players were included in the study	

Kofotolis [24]	- Previous injury - Age - Body mass - Height - Years of training	1. History of previous ankle sprain was a predictor variable in the under 15 age group 2. There was an increased risk of injury for the U15 group versus U12 groups	1. RR = 1.69 95% CI 1.0 to 2.9 2. RR = 2.34 95% CI 1.28 to 5.02	- Body mass - Height Years of training	677 players were monitored for one season (211 were recorded and specifically 38 ankle injuries)

Aoki et al. [39]	- Age - Height - Weight - Playing turf	1. Low back pain showed a significantly higher incidence in the artificial grass versus the natural grass	1. IRR = 1.62 95% CI 1.06 to 2.48 *p* < 0.05	- Older taller, and late adolescent players had a significantly lower incidence of chronic pain	301 players participated to the study (256 injuries were registered on natural turf, 169 on artificial turf)	**TURF TYPE AND EQUIPMENT**

Haag et al. [56]	- Sex - Age - Previous injuries - Body mass index - Training experience - Playing surface - Position Weekly training load	1. Female vs Male 2. U19 vs U15 3. U17 vs U15 4. Previous spine injuries 5. Previous hip/groin injuries 6. Natural vs artificial turf 7. Goalkeeper vs midfielder	1. OR = 1.48 95% CI 1.05 to 2.08 *p* = 0.019 2. OR = 1.84 95% CI 1.21 to 2.80 *p* = 0.004 3. OR = 1.66 95% CI 1.19 to 2.31 *p* = 0.003 4. OR = 1.74 95% CI 1.21 2.52 *p* = 0.003 5. OR = 1.40 95% CI 1.02 to 1.93 *p* = 0.039 6. OR = 1.56 95% CI 1.15 to 2.10 *p* = 0.004 7. OR = 1.70 95% CI 1.04 to 2.78 *p* = 0.036	- Body mass index - Training experience Weekly training load	1110 soccer players were included in the study

Emery & Meeuwisse [60]	- Age - Sex - Playing turf Playing level	1. U14 versus U18 (outdoor turf) 2. Outdoor versus indoor (Division 1) 3. Division 1 versus division 3–4 (outdoor) 4. Division 2 versus division 3–4 (outdoor)	1. RR = 2.73 95% CI 1.39 to 5.77 2. RR = 3.22 95% CI 1.8 to 6.12 3. RR = 5.4 95% CI3.1 to 9.91 4. RR = 2.13 95% CI 0.53 to 5.37	- Sex - Division 1 versus division 3–4 (indoor) Division 2 versus division 3–4 (indoor)	233 players took part in the study and 142 players participated to the indoor comparison study (35 injuries were reported during indoor season and 78 injuries during outdoor season)	**TURF TYPE AND EQUIPMENT**

O’Kane et al. [65]	- Pre-menarche versus post-menarche status - Type of field turf - Type of shoes Playing position	1. Grass field 2. Wear cleats on grass versus wear cleats on artificial turf 3. Defender versus forward	1. OR = 2.83 95% CI 1.49 to 5.31 2. OR = 2.40 9% CI 1.03 to 5.96 3. OR = 1.89 95% CI 1.03 to 4.317	- Pre-menarche versus post-menarche status - Wet surface	351 young female soccer players were followed over 4 seasons (173 acute lower extremity injuries were reported)

Bowen et al. [21]	- Cumulative workloads (1, 2, 3 and 4 weekly) and acute:chronic (A:C) workload rations: • Total distance (TD) • High-speed distance (HSD) • Accelerations (ACC) Training load (TL)	1. High total distance (TD; 112244–143918 m) over 4 weeks (overall injuries) 2. 2Moderate-high 4-weekly high-speed distance (HSD; 3502–5123 m) (non-contact injuries) 3. Moderate-high 1-weekly HSD (856–1449 m) (overall injuries) 4. Accelerations (ACC; ≥ 9254) performed in 3 weeks (overall and non-contact injuries) 5. High 1-weekly load (TL; 474–648 AU) (overall and non-contact injuries) 6. Very high 1-weekly TL (≥ 648 AU) (contact injury) 7. A:C TD (≥ 1.76) (contact injury) 8. Low chronic HSD (< 938 m) and high A:C HSD (1.41–1.96) (non-contact injuries) 9. High chronic HSD (> 938 m) and moderate-high A:C HSD (0.91–1.34) (non-contact injuries) 10. A:C ACC ratio very high (1.77) (contact injuries) 11. Moderate-high A:C TL (0.88–1.32) (non-contact injuries) 12. Moderate-low A:C TL (0.44–0.88) (contact injuries)	1. RR = 1.64 95% CI 1.05 to 2.58 *p* = 0.031 2. RR = 2.14 95% CI 1.31 to 3.50 *p* = 0.003 3. RR = 1.73 95% CI 1.06 to 2.84 *p* = 0.029 4. RR = 3.84 95% CI 1.57 to 9.41 *p* = 0.003 RR = 5.11 95% CI 1.75 to 14.96 *p* = 0.003 5. RR = 1.65 95% CI 1.04 to 2.62 *p* = 0.032 RR = 2.20 95% CI 1.25 to 3.9 *p* = 0.007 6. RR = 4.84 95% CI 1.26 to 18.55 *p* = 0.022 7. RR = 4.98 95% CI 1.31 to 19.02 *p* = 0.019 8. RR = 2.55 95% CI 1.15 to 5.68 *p* = 0.022 9. RR = 2.09 95% CI 1.06 to 4.12 *p* = 0.033 10. RR = 4.98 95% CI 1.30 to 18.99 *p* = 0.019 11. RR = 1.87 95% CI 1.12 to 3.12 *p* = 0.016 12. RR = 1.92 95% CI 1.07 to 3.45 *p* = 0.028	- TD above 143918 m - Low (0–8812 m) TD reduced the risk of overall and non-contact injury - Low 1-weekly HSD (0–756 m) significantly reduced overall and non-contact injury - A low amount of ACC over 3 weeks (744–2861) reduced non-contact and overall injury risk - A low 1-weekly TL (0–130 AU) significantly reduced overall an non-contact injuries - Low chronic TD (< 22335 m) with low A:C TD (0–0.32) reduced overall injury risk - A low ratio (0–0.36) for all chronic HSD significantly reduced the overall injury risk Low A:C ACC (0–0.33) with low chronic accelerations (< 1856) reduced overall injury risk	32 players were monitored through 2 seasons (138 injuries were recorded. 6.9/1000 hours of non-contact injuries 5.2/1000 hours of contact injuries)	**TURF TYPE AND EQUIPMENT**

Bell et al. [67]	- Sport specialization Volume of training per year	1. High sport specialization (Overuse knee injury) 2. High sport specialization (Acute knee injury) 3. Volume of training > 8 months (Overuse knee injury)	1. OR = 2.05 95% CI 1.07 to 3.90 *p* = 0.03 2. OR = 1.68 95% CI 1.01 to 2.78 *p* = 0.046 3. OR = 1.97 95% CI 1.01 to 3.86 *p* = 0.048	- Moderate sport specialization Volume of training > 8 months (Acute knee injury)	761 young soccer athletes were included in the study

Frome et al. [68]	- Age - Sport specialization - Training ratio between weekly hours in organized sports and weekly hours in recreational free play	1. Age 2. Sport specialization (severity) 3. Training ratio > 2	1. OR = 1.10 95% CI 1.04 to 1.16 *p* < 0.01 2. IQR = 2–4 *p* = 0.0003 3. OR = 1.35 95% CI 1.15 to 1.59 *p* = 0.0003	- Specialized soccer athletes had decreased odds of reporting at least one previous injury compared with non-specialized athletes and similar odds of reporting at least one previous lower extremity overuse injury	2099 elite male youth soccer players were included in the study	**EXTERNAL WORKLOAD AND SPORT SPECIALIZATION**

Bacon & Mauger [54]	- Total distance (TD) • High-speed running meters (HSR)			- Total distance (TD) - High speed running meters (HSR)	41 youth soccer players were followed over 2 seasons (85 overuse injuries were recorded)

b = effects; CI = confidence interval; d = Cohen’s d effect size; ES = effect size; HR = hazard ratio; IQR = interquartile range; IR = injury risk; IRR = injury rate ratios; OR = odds ratio; P = significative level; r = Pearson correlation; RR = relative risk.

Three studies [7, 33, 54] evaluated aerobic fitness of the players. Two studies [7, 33] did not find any link with injuries, while Watson et al. [55] observed that players’ lower preseason aerobic fitness levels were associated with a higher risk of subsequent in-season injury and illness.

Clausen et al. [63] identified the KOOS questionnaire, composed of different subscales (function in daily living, pain subscale, function in sport and recreation, knee-related quality of life subscale, other symptoms) as a sensitive tool to identify players who were more prone to injuries. One study [64] analysed the impact of psychological factors on injuries. The authors found high levels of perceived life stress, high levels of perceived mastery climate, and high levels of life event to be psychological risk factors.

### Biological risk factors

Within this section, we grouped injury risk factors related to chronological age [7, 20, 24, 33, 34, 39, 45, 50, 55–60, 62, 68, 80], biological age [20, 23, 35, 38, 43, 46, 66, 86], sex [33, 50, 56, 59, 60] and menarche status [48, 58, 65].

Ten studies [20, 24, 50, 56–58, 68, 80, 81, 85] identified advanced chronological age as an injury risk factor, whereas seven studies [7, 34, 45, 55, 59, 62, 64] did not find significant differences, while the remaining articles [33, 39, 60] reported a higher risk in younger soccer players.

Seven studies [28, 35, 38, 43, 46, 66, 86] investigated injury risk according to biological maturity. Most of these studies applied the Mirwald et al. [89] algorithm to assess the peak height velocity (PHV) used as an indicator of maturity timing. Only three studies [28, 46, 86] determined skeletal age using hand-wrist radiographs. Two studies [35, 66] found a higher injury risk during the PHV time compared to the period before the PHV, while Van der Sluis et al. [38] identified at-risk players as those who presented their PHV at an older age. Bult et al. [43] detected the six months after the PHV as more critical for injury risk. Among the studies assessing skeletal age, Johnson et al. [46] and Materne et al. [86] adopted the Fels method, while Le Gall et al. [28] used the Greulich–Pyle method. Le Gall et al. [28] found a higher incidence of tendinopathies, groin strains, and re-injuries in early-maturing players. Similarly, Materne et al. [86] identified a significantly greater risk of injury in early-maturing players compared with normal and mature players. In contrast, Johnson et al. [46], did not identify significant differences according to skeletal age when mean playing time, mean training time, mean height, and position played were considered in the analysis. Read et al. [23] assessed PHV only to analyse how biological maturity affects neuromuscular control. The authors observed higher landing forces on the left leg in players during PHV and lower knee valgus in players after the PHV period. Likewise, three studies [48, 58, 65] investigated the impact of menarche status on neuromuscular control in young female soccer players. O’Kane et al. [48] identified post-menarche status as a risk factor, while the remaining studies [58, 65] did not find significant differences between pre-menarche and post-menarche status.

Regarding sex differences, one study [56] found a higher injury risk in young female soccer players, whilst four studies [33, 50, 59, 60] did not identify any significant difference.

### Anthropometric risk factors

Seventeen studies [7, 24, 25, 33, 34, 39, 45, 55–59, 62, 64, 72, 83, 87] discussed anthropometric risk factors.

Most studies did not identify height [24, 33, 34, 57, 62, 64] or body mass [7, 24, 33, 34, 45, 55, 59, 62, 64] as risk factors. Likewise, body mass index was not associated with injuries in young soccer players [7, 33, 34, 45, 56, 58, 59, 62, 64].

However, Venturelli et al. [7] found an association between height and muscular strains. Moreover, Bastos et al. [45] and Rössler et al. [59] observed that taller players reported more injuries than shorter ones. Kemper et al. [25] investigated the monthly changes in height and body mass index. The authors found that growth rates of at least 0.6 cm/month, monthly BMI-increase of > 0.3 kg/m^2^ and decrease in BMI value of at least 0.4 kg/m^2^ were potential risk factors. Similarly, Rommers et al. [87] found a 15% increase in injury risk per cm of growth per year. Moreover, Rommers et al. [83] identified a greater increase in leg length (cm/year) as an overuse injury risk factor in young soccer players. One study [72] examined hamstring length without finding an association with injuries.

Only two articles [57, 83] reported weight and body mass index as injury risk factors.

### Previous injuries

Eleven studies [7, 24, 27, 33, 41, 56–58, 62–64] analysed previous injuries as an intrinsic risk factor.

Most of them [7, 24, 33, 41, 56, 62–64] found a strong association between injury history and the risk of new injuries. Moreover, Kucera et al. [41] showed that the risk increased in players with two or more previous injuries (RR = 2.97) compared to players with one previous injury (IRR = 2.57). One study [58], even though not identifying an association between previous injuries and new injuries, found a familiar disposition to ACL injury as a risk factor.

Two studies [27, 57] did not identify injury history as an intrinsic risk factor.

### Technical and tactical skills

Only two studies [26, 29] investigated the impact of technical and tactical skills on the risk of injury. Both these studies agreed that skilled players have a higher risk of injury. Particularly, Soligard et al. [26] observed that players with good ball-receiving, highly skilled in passing, shooting, heading, tackling, and dribbling were exposed to a higher risk of injuries. The authors assessed tactical abilities as well, and they observed that players who made good decisions when in ball possession and in defence incurred a significantly higher risk of injuries.

Schwebel et al. [29] also confirmed that skilled players were at higher risk of injuries. However, even less experienced players presented a greater predisposition to be injured.

### Extrinsic risk factors

Extrinsic risk factors are defined as factors that are outside the body, irrespective of the injured players, and closely linked to the type of activity practised [88]. A total of seventeen articles [19, 21, 24, 39, 44, 45, 54, 56–60, 62, 64, 65, 67, 68] investigated injury extrinsic risk factors.

### Turf type and equipment

Seven studies [19, 39, 56, 58–60, 65] reported the impact of the type of turf on injuries. Haag et al. [56], as well as O’Kane et al. [65] found a higher injury risk playing on natural turf than artificial turf. Conversely, Rössler et al. [59] observed a higher predisposition to injury playing on artificial turf (RR = 1.39). Similarly, Aoki et al. [39] reported a higher incidence of low back pain in players who trained on artificial grass. On the other hand, Emery & Meeuwiss [60] recorded a higher incidence of injuries in outdoor turf compared to indoor turf (RR = 3.22). One study [58] did not find significant differences between artificial turf and natural grass.

One study [19] prospectively investigated injury incidence in young soccer players who played on artificial turf, but without a direct comparison with injuries that occurred on natural grass.

Moreover, one study [65] examined the impact of the type of shoes on injury risk. The authors found that wearing cleats on grass produces a higher risk of injuries compared to cleats worn on artificial turf (OR = 2.40).

Instead, Lukášek & Kalichová [44] investigated the head acceleration during impact with the ball.

### External workload

Seven articles [21, 54, 57–59, 62, 67] studied the association between external workload and injuries in young soccer players. Two studies [21, 54] prospectively collected GPS data, relating them to injury incidence. Bowen et al. [21] analysed the cumulative workload and the acute:chronic (A:C) workload ratio of total distance (TD), high-speed distance (HSD), and accelerations (ACC). These authors found that a high cumulative workload in 1 and 4 weeks, and a high A:C ACC, were associated with a greater injury risk, as reported in Table 2. Likewise, Bacon & Mauger [54] identified an association close to significant levels between the total distance and high speed running cumulative load of 2 weeks with overuse injuries. Three studies [57, 58, 62] evaluated weekly training exposure, but the authors did not identify hours of training per week or training sessions per week as risk factors. Bell et al. [67], however, observed that an annual training volume greater than 8 months produced a higher risk of overuse knee injuries. Rössler et al. [59] investigated the match-training ratio, observing that adolescent players who had more training exposure suffered from fewer match injuries. Indeed, non-injured players presented a match-training ratio of 1:7, unlike injured players, who had a match-training ratio of 1:5.

### Playing position and sport specialization

Several studies [24, 56, 57, 59, 65] investigated how different playing positions may elicit different injury risks. Kofotolis [24] reported a higher injury rate in goalkeepers compared with forwards and defenders. Similarly, Haag et al. [56] found a higher risk of injury in goalkeepers compared to midfielders (OR = 1.56). Meanwhile, O’Kane et al. [65] observed that defenders were more exposed to injury risk compared with forwards (OR = 1.89). On the other hand, two studies [57, 59] did not identify playing position as a risk factor. Moreover, Sugimoto et al. [57] observed that players who experienced multiple playing positions reported a history of injuries with fewer musculoskeletal strains compared to single-position players.

Regarding this topic, sport specialization, defined as intense practice in one sport while excluding others [90], was investigated in seven articles [24, 45, 56, 62, 64, 67, 68]. Bastos et al. [45] found that players with more than five years of training were more prone to injuries compared to players with less than five years of training. Steffen et al. [62, 64] obtained similar results. The authors found that the risk of injury rose with increasing years of organised soccer play. Consistently with these results, Bell et al. [67] noted that highly specialised athletes reported more overuse and acute knee injuries. However, Frome et al. [68] did not find an association between sport specialization and risk of injury; also Kofotolis [24] and Haag et al. [56] did not identify the number of years of training as an injury risk factor.

## DISCUSSION

The aim of this review was to develop an overall point of view of the potential risk factors in young soccer players and to discuss how these factors could interact and determine the injury onset.

Different intrinsic (neuromuscular control, chronological age, biological age, sex, previous injuries, technical and tactical skills) and extrinsic (turf type, external workload, playing position, and sport specialization) injury risk factors were identified and discussed in the subsequent sections.

The identification of injury risk factors represents the step immediately preceding the implementation of prevention strategies [13]. This process is crucial in young soccer players to promote proper talent development, but at the same time, it is extremely complex. Indeed, injury is a multifactorial phenomenon determined by the interaction of numerous elements. These elements are represented by the injury risk factors, which interacting with each other result in a “web of determinants” generating unpredictable and unplanned relations [91]. According to the Meeuwisse’s dynamic model [14], the combination of these factors (intrinsic and extrinsic) makes athletes prone to injury. However, all these factors together represent the necessary but not sufficient condition for causing an injury, because the presence of an inciting event (e.g. match schedule, playing situation, joint motion) is the real factor that determines the onset of the injuries [92].

Faced with a complex phenomenon such as injury, many authors used a reductionist approach, which allows analysis of the various risk factors separately [91]. This approach is useful in simplifying the interpretation of the phenomenon, but does not allow the analysis of how the interaction of different factors may determine the onset of injuries.

### Intrinsic risk factors

Several intrinsic risk factors were identified in the current review: neuromuscular control, physiological and psychological factors, biological and anthropometric factors, previous injuries, technical and tactical skills.

Almost half of the articles selected in this review investigated the link between alteration in neuromuscular control and injury risk. Neuromuscular control is defined as the unconscious response of a muscle to a stimulus to ensure dynamic joint stability [93]. This means that a deficit in the neuromuscular control may produce excessive joint stress [94] leading consequently to an injury. Different screening tests were adopted in the scientific literature to detect neuromuscular dysfunction. Ko et al. [34] identified an association between a low score in the star excursion balance test (SEBT) and risk of ankle sprain.

Four studies investigated [47, 47, 61, 82] knee control during a motor task. Räisänen et al. [47] did not find an association between the FKPA during single-leg squat and injuries, while O’Kane et al. [48, 61] identified knee valgus as an intrinsic risk factor. The conflicting results may be explained by the different evaluation methods (FKPA vs NKS) and different motor tasks (single-leg squat vs drop-jump test) used. However, knee valgus seems to be a risk factor only in female postmenarchal players [48], confirming the idea that an injury is determined by the interaction of different factors. Puberty is a critical period for young athletes, characterised by rapid changes in stature, limb flexibility, strength and in hormonal profile [95]. Indeed, Nguyen et al. [49], investigating longitudinal changes in hip strength and range of motion in female youth soccer players, found an increase in hip internal rotation and abduction with a contemporary decrease in external rotation and adduction. The changes in hip range of motion may alter the neuromuscular control, compromising muscular activation during dynamic activities, and consequently increasing lower extremities’ injury risk [49]. Moreover, the higher oestrogen concentration during the postmenarchal period may affect the ligament structures and explain the greater predisposition to knee injuries in female athletes [95]. Although some studies confirmed a higher injury predisposition during adolescence in female soccer players [56], puberty represents a sensitive time also in young male soccer players. A few studies [28, 46, 86] used hand-wrist radiographs to investigate the impact of maturity status on injury risk. Materne et al. [86] and Le Gall et al. [28] found a higher risk of injury in early-maturing players. Instead, the most common method to assess the maturity status was the Mirwald et al. [89] algorithm, performed to determine the peak height velocity (PHV) of the young soccer players. Although the the maturity offset can present an error of ± 1 year 95% of the time [89], it is a very useful tool because it is not invasive. The period around PHV was identified as critical in the increase of injury risk [35, 43, 66]. During this phase, players experience rapid growth, and changes in muscle-tendon junctions, ligament, cartilage, and bone density [35]. This increase in the vulnerability of muscular tissues, associated with a concomitant rise in training load, may explain the higher injury incidence in the PHV phase. Furthermore, this stage has been defined as a period of “adolescent awkwardness” in which there is impairment in motor skill performance due to the onset of rapid growth processes [96]. In support of this hypothesis, Read et al. [23] found higher landing forces on the left leg in players during PHV and lowering of knee valgus in players after PHV. The findings of this study show that high landing forces may be associated with greater injury risk and may be due to the temporary decrease in motor skills [23].

During a soccer match, the neuromuscular control may also be altered by the players’ state of fatigue due to the increase in minutes of play. As proof of this, several studies [75, 76, 78, 79, 97–100] reported higher injury incidence during the second half of the match, in particular during the last 15 minutes. Different studies tried to replicate soccer-specific fatigue to investigate its effect on neuromuscular control. Lehnert et al. [30, 52] observed a decrease in absolute and relative leg stiffness after SAFT protocol execution, while De Ste Croix et al. [22] reported a longer electromechanical delay (EMD) in U13 soccer players. The impairment of these mechanisms may be dangerous for joint stability. Indeed, the reduction in leg stiffness is associated with greater ground contact time, changes in centre of mass displacement, and consequently less movement efficiency and increase in shear force absorption [52]. Likewise, a longer EMD reflects an alteration in muscle activation. However, the fatigue produced through the SAFT protocol seems to have no effects on iso-kinetic strength of the hamstrings and quadriceps [30], on hamstring/ quadriceps ratio [30, 52], or on knee kinematics registered during a single leg drop jump and countermovement jump [71]. Meanwhile, Wollin et al. [51] investigated the effect of fatigue produced by a congested soccer match period. The authors found a transient reduction in hamstring strength and associated pain during hamstring maximum voluntary isometric contraction. These results highlight an incomplete recovery that may persist for up to 48 hours after a match and the need to monitor players’ physiological responses to avoid the risk of injuries.

All these studies investigated the effect of fatigue on physiological parameters in a short time period, i.e. after SAFT protocol execution [22, 30, 52, 71] or after a congested match period [51], without being able to study the association with the onset of the injuries. For this purpose, different studies [5, 31, 69] investigated prospectively the stress and fatigue state induced by training, and the link with injuries during one [31, 69] and two seasons [5]. Brink et al. [5], as well as Watson et al. [69], found an association between high internal training load (s-RPE) [5, 69], monotony, and strain [5] with injuries and illness. These results highlight the need to monitor stress and recovery to identify young soccer players at risk of injuries and illnesses. Moreover, physical trainers and coaches should be aware of the importance of improving the physical fitness of the athletes needed to tolerate a high training load during the season. Indeed, Watson et al. [55] found that lower preseason aerobic fitness was associated with a higher risk of injuries and illness during the season. However, Raya-González et al. [31] did not find any association between weekly training load and risk of injury. This discrepancy may be explained by the small sample size and the small number of injuries found.

In addition to physiological responses, other authors [63, 64] considered it important to monitor the psychological sphere. Indeed, Steffen et al. [64] identified a high level of perceived life stress, high levels of perceived mastery climate and high levels of life event as injury risk factors. Therefore, coaches must be able to create a positive motivational atmosphere, reducing players’ life stress perception. In fact, stress may increase muscle tension and impair motor control [101], causing a higher predisposition to injury.

Among the several intrinsic risk factors analysed, the anthropo-metric parameters were widely investigated. Most studies agree that neither height [24, 33, 34, 57, 62, 64] nor body mass [7, 24, 33, 34, 45, 55, 59, 62, 64] nor body mass index [7, 33, 34, 45, 56, 58, 59, 62, 64] is associated with higher injury risk. Rather, it was found that a rapid gain in height or body mass index represented an injury risk factor in itself [25, 83, 87]. These results confirm the problems, previously mentioned, related to the growth process that occurs around PHV. A few studies recognised stature as an injury risk factor [7, 45, 59]. In this case, the authors tried to explain these results through the higher biomechanical load [59] or the poor motor coordination [7] that characterizes taller players. However, while poor motor skills could be an injury risk factor [7, 96], two studies [26, 29] also identified highly skilled players to be at risk of injury. However, this should not be misunderstood. Indeed, the authors simply suggest that skilled players, with good technical and tactical abilities, are more involved in the game and consequently more exposed to tackles and duels. Therefore, high motor skills do not represent a direct risk factor, but rather expose athletes to contact and duels. For this reason, it is important to promote a fair-play policy and encourage proper rule enforcement and adherence [102, 103].

Aware of the several factors that may lead to an injury, it is important to have an overview of the risks connected with playing soccer, to promote prevention strategies and to reduce the onset of injuries. This is crucial, because an injury may represent in turn an intrinsic risk factor for new injuries. Many studies [7, 24, 27, 33, 41, 56, 62–64] investigated the association between injury history and new injuries.

Most of them [7, 24, 33, 41, 56, 62–64] found a strong relationship, with the risk increasing with the number of previous injuries [41].

### Extrinsic risk factors

In the present review, turf type, equipment, external workload, playing position, and sport specialization were classified as extrinsic risk factors.

Several studies investigated the impact of the playing surface on the injury risk. The related results are controversial and difficult to interpret. Two studies [56, 65] found a higher injury risk playing on natural turf compared to artificial turf. Conversely, Rössler et al. [59] and Aoki et al. [39] reported a higher risk on artificial turf. Moreover, one study [58] did not find differences between the playing surfaces. A meta-analysis tried to clarify the discrepancy of the results [104]. The authors found a lower injury incidence rate playing on artificial turf. However, considering the heterogeneity of the studies included in the meta-analysis, it is difficult to reach an absolute conclusion. Even in the present review, the selected studies are characterised by different experimental designs. Moreover, we must consider the quality and generation of the fields employed in the studies. It often happens that young soccer players train on worn playing surfaces, unlike elite and adult soccer players [59]. In addition to the playing surface, the type of shoes may represent an extrinsic risk factor. Unfortunately, only one study [65] approached this issue. The authors observed that wearing cleats on grass was associated with a higher risk of injuries compared to wearing cleats on artificial turf.

Lukášek & Kalichová [44] stated that repeated head impacts with the ball may be dangerous in young soccer players, causing a functional problem in the brain. In light of this statement, recently the Scottish Football Association decided to ban heading for under 12 players. However, the article mentioned presents a low-quality score.

Even exposing athletes to high training volume may be dangerous [21, 54, 67]. As previously mentioned, young soccer players experience a period of rapid changes in muscle, tendon, and ligament structures, and a concomitant increase in the training volume may lead to a greater predisposition to injury. Bowen et al. [21] and Bacon & Mauger [54] observed that higher cumulative workload in total distance, high-speed distance, and accelerations was associated with greater injury risk. These results, according to the Kenttä & Hassmén [105] model, suggest that a higher workload, associated with poor recovery status, increases the risk of overuse injuries. In confirmation of this, Bell et al. [67] reported that playing or training in soccer for more than 8 months per year increased the risk of overuse injuries. This does not mean that it is wrong to promote intense training in young soccer players; a proper training stimulus increases physical tolerance and resilience to injury risk [21]. Indeed, Rössler et. [59] observed that a greater weekly training volume had a protective effect on injuries. Rather, it is important to ensure adequate recovery and to avoid a sudden increase in the weekly workload, as suggested by Gabbet [106]. Moreover, coaches and trainers must be able to adapt the physical demands in relation to the individual differences (e.g., sex, age, maturity status). These physical demands may also change in relation to playing position. Indeed, coaches and physical trainers should be aware that different playing positions elicit different physical efforts and different involvement in the game, and consequently different exposure to injury. O’Kane et al. [65] found higher injury risk in defenders, while Haag et al. [56] found higher injury risk in goalkeepers. It is difficult to reach a conclusion from these results, because different play styles and strategies, competitive levels and different skill levels may influence the risk of injury. In addition, Sugimoto et al. [57] observed that players who experienced multiple playing positions reported fewer musculoskeletal injuries compared to single-position players. We can speculate that experiencing different playing positions may increase adaptability to distinct tasks and improve technical and tactical skills. On the other hand, the specialization in a single playing position may subject athletes to the same repetitive movement and increase the risk of overuse injuries. This aspect is closely linked to the problem of early specialization in young athletes. Several studies [45, 62, 64, 67] reported an association between soccer specialization and injury risk. Moreover, other factors such as pressures placed on the athletes could lead to stress, lack of energy, sleep disturbances, and consequently burnout [107].

For this reason, it is important to promote multisport participation to avoid overuse injuries as well as to improve decision-making skills and mental health and to encourage social relationships with other peers.

## STRENGTHS AND LIMITATIONS OF THE REVIEW

According to our knowledge, this is the first review that has tried to summarize the injury risk factors in young soccer players. However, there are several limitations to consider. As in our companion review (part I) [108], the heterogeneity of the studies, mainly due to different experimental designs and different injury risk investigated, did not allow us to perform statistics or meta-analysis of the results.

Several studies, classified as cross-sectional or quasi-experimental studies [22, 23, 30, 40], reported injury risk factors; however, the authors did not investigate the direct association with the onset of injuries. Therefore, the risk factors were included in the present review, but a longitudinal design is needed to confirm a possible association.

The injury risk factors were grouped into intrinsic and extrinsic risk factors; however, the use of different tests, collection processes, follow-up period, and the different injury types investigated (e.g., overuse, traumatic, non-contact) make comparison between studies difficult. Moreover, all the risk factors were discussed together in the present review, albeit collected on samples characterised by different age and sex. The publications are limited to English language, and relevant studies may have been excluded.

## CONCLUSIONS

Injury is a complex and multifactorial phenomenon. A deep awareness of these factors is crucial to promote adequate prevention strategies. In the present review, injury risk factors were divided into intrinsic and extrinsic ones. Among the intrinsic risk factors, the following results have been reported:

– Proper neuromuscular control plays an important role in limiting the risk of injury. Indeed, limb asymmetry and knee valgus on landing were identified as relevant injury risk factors.
– Maturation processes may increase the injury risk in postmenarchal female players and in male soccer players during PHV.
– Physiological factors, such as fatigue and poor recovery, may contribute to the increase in injury risk.
– The results related to anthropometric characteristics (i.e., height and body mass) are still controversial.
– Previous injuries were recognised as important intrinsic factors.
– Other studies, investigating the impact of technical and tactical skills, identified highly skilled players as being at greater risk of injuries.

Among extrinsic risk factors, the following results have been reported:

– The results related to playing surface are controversial.
– An excessive weekly workload increased the risk of injuries. The risk could also be linked to the playing position, but the results analysed in the present review are unclear.
– Some authors found that sport specialization and a high annual volume of training increased the risk of overuse and acute injuries.

Future studies should aim to clarify how the injury risk changes in relation to chronological age, maturation, and sex. Moreover, it would be useful to develop a test battery to identify players at risk of injury. Considering the various factors linked to the onset of injuries, authors should promote longitudinal investigations with a complex system rather than a reductionist approach.

## Funding

The authors received no specific funding for this work.

## Conflicts of interest/Competing interests

The authors have declared that no conflicts/competing interests exist.

## Contributorship

MM and AT was responsible for the conception and design of the study. MM, AT and AF conducted the literature review. MM, AT and MG contributed to data collection and interpretation. The article was written by MM and AT. All authors contributed to the reviewing of the manuscript.
